# Live visualization of genomic loci with BiFC-TALE

**DOI:** 10.1038/srep40192

**Published:** 2017-01-11

**Authors:** Huan Hu, Hongmin Zhang, Sheng Wang, Miao Ding, Hui An, Yingping Hou, Xiaojing Yang, Wensheng Wei, Yujie Sun, Chao Tang

**Affiliations:** 1 School of Life Sciences, Peking University, Beijing 100871, China; 2 Center for Quantitative Biology, Peking University, Beijing 100871, China; 3 Peking-Tsinghua Center for Life Sciences, Peking University, Beijing 100871, China; 4 Biodynamic Optical Imaging Center (BIOPIC), Peking University, Beijing 100871, China; 5 Academy for Advanced Interdisciplinary Studies, Peking University, Beijing 100871, China; 6 Beijing Advanced Innovation Center for Genomics (ICG), Peking University, Beijing 100871, China; 7 State Key Laboratory of Protein and Plant Gene Research, Peking University, Beijing 100871, People’s Republic of China

## Abstract

Tracking the dynamics of genomic loci is important for understanding the mechanisms of fundamental intracellular processes. However, fluorescent labeling and imaging of such loci in live cells have been challenging. One of the major reasons is the low signal-to-background ratio (SBR) of images mainly caused by the background fluorescence from diffuse full-length fluorescent proteins (FPs) in the living nucleus, hampering the application of live cell genomic labeling methods. Here, combining bimolecular fluorescence complementation (BiFC) and transcription activator-like effector (TALE) technologies, we developed a novel method for labeling genomic loci (BiFC-TALE), which largely reduces the background fluorescence level. Using BiFC-TALE, we demonstrated a significantly improved SBR by imaging telomeres and centromeres in living cells in comparison with the methods using full-length FP.

Accumulating evidence has revealed that the structure of the mammalian genome is organized and regulated in space and time^1^,^2^,^3^,^4^, accompanying development, cell proliferation, and cell differentiation^5^,^6^, while disorder of nuclear reorganization can lead to some human diseases^7^. To study the spatiotemporal organization of chromosomes, it is crucial to develop tools for the visualization of specific genomic loci.

Fluorescence *in situ* hybridization (FISH) has been widely used to label specific genomic loci, but it requires DNA denaturation and fixation of the cell, which is incompatible with live-cell imaging. Recently, a new approach, dCas9-mediated fluorescence *in situ* hybridization (CASFISH), has been developed to label loci without DNA denaturation^8^. In order to label the loci in living cells, DNA-binding proteins and the fluorescent repressor and operator system (FROS) have been used^9^. However, DNA-binding proteins usually require specific DNA sequences, while applications of FROS in mammalian cells are constrained due to inefficient loci-specific targeting. Over the past few years, several new strategies have been developed based on genetically engineered protein modules that recognize DNA sequences in a specific manner. TALEs were originally discovered from the plant pathogenic bacteria *Xanthomonas* and they can bind to target DNA sequences through ~34-aa repeats. Each such repeat can recognize a specific single base pair through two adjacent amino acids, called repeat-variable diresidues (RVDs)^10^,^11^. The most widely used CRISPR system derived from *Streptococcus pyogenes* is the type II CRISPR/Cas9 system and it uses a small guide (sg) RNA to help a Cas9 protein to recognize DNA sequences^12^,^13^. Particularly, both the transcription activator-like effector (TALE)^14^,^15^ and the clustered regulatory interspaced short palindromic repeats (CRISPR/Cas) systems^16^ have been shown to be promising tools for live-cell genomic fluorescent labeling. Further development of these systems could help answer many fundamental questions that await such kind of tools. One of the critical goals is to label multiple gene loci with multiple colors, so we and other groups have recently developed several such techniques in living cells^17^,^18^,^19^,^20^. Another critical goal is to label non-repetitive, single-gene loci using a minimal number of probes. To this end, ultra-sensitive detection implemented by increasing the signal-to-background ratio (SBR) is extremely important. For cellular imaging, a major source of background fluorescence is the diffusing fluorescent probes, which hamper the application of the current methods based on TALE and CRIPSR/Cas9.

In this work, we set out to develop a new genomic labeling method by combining bimolecular fluorescence complementation (BiFC) with TALEs in order to reduce the background fluorescence. BiFC is based on the structural complementation between two non-fluorescent N-terminal and C-terminal fragments of a fluorescent protein^21^, which makes this system an ideal tool for suppressing background fluorescence in the labeling and imaging of genomic loci^21^,^22^,^23^,^24^,^25^,^26^. In comparison with the existing methods, which are all based on full-length fluorescent proteins (FPs), the BiFC-TALE imaging system eliminates the background fluorescence from diffuse FPs. With BiFC-TALE, the SBR is >8-fold higher than in the TALE method with full-length FP.

## Results

### Development and optimization of the BiFC-TALE system

**Figure 1:**
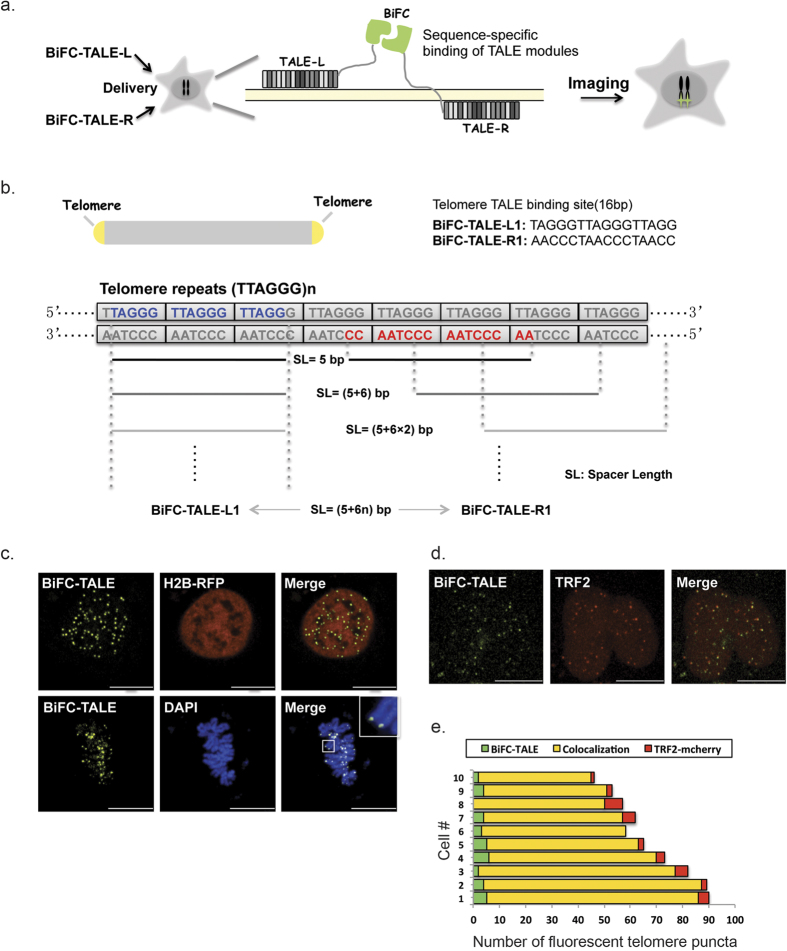
Schematic of BiFC-TALE for genomic sequence imaging and its application in visualizing telomeres in living human cells. (**a**) Overview of the imaging system. Sequence-specific BiFC-TALE fluorescent signals allow the imaging of genomic loci in living cells. (**b**) Upper panel, TALE targeting sequences of telomeres. Lower panel, spacer length within the BiFC-TALE-L1/R1 pair for telomeres: the spacer length of this pair is 5 + 6n bp on the DNA. Pairs of gray lines show three possibilities for spacer length within BiFC-TALE-L1/R1: when BiFC-TALE-L1 was fixed to its targeting sequence (blue nucleic acids), BiFC-TALE-R1 could be shifted to different binding sites (red nucleic acids). (**c**) BiFC-TALE images of telomere sequences in interphase and mitotic phase HeLa cells: telomeres labeled with BiFC-TALE-L1/R1 (yellow); nuclei stained with H2B-RFP (red) or DAPI (blue). Scale bars, 10 μm. (**d**) Examples of co-labeling of telomeres using BiFC-TALE (yellow) and the telomere-binding protein TRF2-mCherry (red). Scale bars, 10 μm. (**e**) Quantification of telomeres targeting-specificity based on the BiFC-TALE and TRF2 channels in 10 exampled HeLa cells.

To establish and optimize the BiFC-TALE system (Fig. 1a), we labeled human cell telomeres, which are composed of 5 to 15 kb tracts of TTAGGG repeats^27^ (Fig. 1b). For the BiFC, we used the C-terminal fragment (VC155) and the N-terminal fragment (VN173)^28^ split from mVenus fluorescent protein. We first fused these fragments to the C-terminus of a pair of TALE modules designed such that they bound head-to-head to the target sites on different strands of the double-stranded DNA. Thus, their fused non-fluorescent fragments were brought together to form an intact fluorescent protein to visualize specific genomic loci in living human cells (Fig. 1a and Supplementary Fig. S1a). We used 2 × GGGGS as the linker between TALEs and non-fluorescent fragments to provide split fluorescent protein fragments sufficient freedom to collide with each other. Since a reconstituted single fluorescent protein is 2–3 nm long and one 2 × GGGGS linker is ~3.8 nm, the spacer length (SL) within a BiFC-TALE pair was <10 nm to ensure complementation of the BiFC fragments.

In total, we designed 3 left and 2 right TALE modules, which yielded 6 L+R combinations (Supplementary Table S1, S2). Due to the repetitive sequences in the telomere, there were many possible spacer lengths for one specific BiFC-TALE pair. Therefore, the 6 combinations corresponded to different patterns of spacer length (3 + 6n, 4 + 6n, 5 + 6n, 6 + 6n, and 7 + 6n), where 6n represents the shift period of the TALE binding sites (Fig. 1b and Supplementary Table S2). To evaluate the performance of the 6 BiFC-TALE combinations, each was expressed in HeLa cells and imaged 24 h post-transfection. Many and clear puncta were observed in cells transfected with the L1/R1 as well as the L3/R1 pair, with much fewer puncta in other groups. All the subsequent telomere labeling and imaging experiments were performed with the BiFC-TALE-L1/R1 pair. Given that the spacer length within BiFC-TALE pair is <10 nm to ensure BiFC, we inferred that the optimal spacer length within the BiFC-TALE-L1/R1 pair was ~11–23 bp (SL = 5 + 6n, n = 1–3), which is ~3–8 nm in DNA length (1 bp = 0.34 nm) for the BiFC-TALE system to work. Although some spacer lengths in other BiFC-TALE combinations for telomeres could also theoretically lie within the range, their evident low labeling efficiency might have been due to their low DNA binding affinity compared to the BiFC-TALE-L1/R1 pair. Actually, such differing affinity of different TALEs has been reported using an algorithm^29^, which, in our hands, showed that L1 and R1 were the optimal TALE modules for the BiFC labeling of telomeres. It should also be noted that TALE repeats with the HD RVD are sensitive to methylation at cytosines, so the DNA methylation on of target sequences could affect the binding affinity of TALEs^30^.

To further testify our assumption that DNA binding affinity of TALE modules can affect the labeling efficiency of our BiFC-TALE system, we used FP-TALE system to evaluate the DNA binding affinity of our telomere TALE modules (Supplementary Table S1 and Supplementary Fig. S2). The result turned out that the labeling of telomeres puncta labeled with FP-TALE-L1, FP-TALE-L3 or FP-TALE-R1 is more efficient than the others, which was consistent with the performance of the 6 BiFC-TALE combinations. Using BiFC-TALE-L1/R1 plasmids, telomeres of HeLa cells were labeled in both interphase and mitotic phase (Fig. 1c), with evident puncta appearing specifically at the ends of mitotic chromosomes. Next, we tracked these BiFC-TALE labeled telomere loci in mitotic HeLa cells (Supplementary Movie S1). To further verify the specificity and efficiency of telomere labeling by our system, we performed two-color imaging with either telomere-specific FISH using Cy5-tagged probes or fluorescent protein fused TRF2, a telomeric DNA repeat-binding protein (Supplementary Fig. S1b and Fig. 1d). Because the FISH procedure denatures DNA, resulting in the dissociation of TALEs and diminishing the BiFC-TALE signal, we first imaged BiFC-TALE before FISH labeling and imaging. The numbers of telomere puncta labeled by both BiFC-TALE and FISH were counted in each cell (Supplementary Fig. S3a,b). Co-localization of the BiFC-TALE and FISH puncta was analyzed using MatLab (Supplementary Fig. S1b, for details see methods). Quantitative analysis of cells co-labeled with BiFC-TALE and TRF2 showed >80% co-localization (n = 53), with 10 examples of them presented (Fig. 1e), indicating comparable efficiency and specificity.

To test the universality of our imaging system, we also introduced the BiFC-TALE-L1/R1 pair to human breast cancer cell line MBA-MD-231 and human retinal pigment epithelium (RPE) cells, and telomeres were successfully labeled in both (Supplementary Fig. S4). Taken together, these data indicate that our approach enables efficient and specific labeling of endogenous repetitive sequences in living human cells.

### Visualization of centromeric alpha-satellite sequences with the BiFC-TALE system

**Figure 2:**
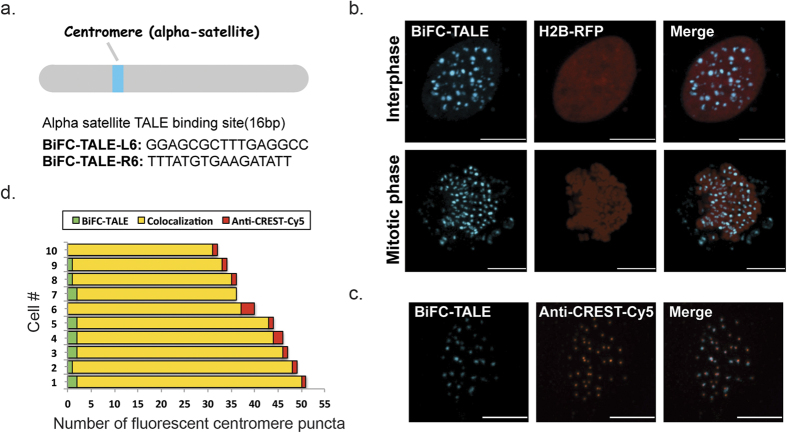
BiFC-TALE system for visualizing centromeric alpha-satellites in living human cells. (**a**) TALE targeting sequences of alpha-satellite. (**b**) BiFC-TALE images of alpha-satellite sequences in interphase and mitotic phase HeLa cells: centromeres labeled by BiFC-TALE-L6/R6 (cyan); nuclei stained with H2B-RFP (red). Scale bars, 10 μm. (**c**) BiFC-TALE labeled alpha-satellite puncta in HeLa cells by immunofluorescence: images for BiFC-TALE (cyan, left), anti-CREST-Cy5 (red, middle), and merged (right). Scale bars, 10 μm. (**d**) Quantitative analysis of centromeric alpha-satellite-targeting specificity by BiFC-TALE with IF in 10 exampled HeLa cells.

To address whether our BiFC-TALE system can be applied to the visualization of other repetitive genome sequences generally, and to test the principles learned from telomere labeling, we designed TALEs that targeted centromeric alpha-satellite repeats and fused them to split mCerulean fluorescent protein C-terminal (CC155) and N-terminal (CrN173) fragments. Alpha-satellite sequence are from a family of AT-rich human centromeric satellites, containing 171-bp repeats^31^,^32^. According to the previously optimal spacer length range, three pairs of BiFC-TALE plasmids for satellites were constructed with different binding sequences (Fig. 2a, Supplementary Fig. S1 and Supplementary Table S1, S2) and then transfected into HeLa cells. The HeLa cells expressing BiFC-TALE-L6/R6, with a spacer length of 15 bp, showed much better satellite puncta imaging than the other two pairs. DNA binding affinity of these alpha-satellites TALE modules was also tested with FP-TALE system (Supplementary Table S1 and Supplementary Fig. S5), turning out the labeling of alpha-satellite puncta labeled with FP-TALE-L6 or FP-TALE-R6 is more efficient than others. With BiFC-TALE-L6/R6, alpha-satellites were also labeled in both interphase and mitotic phase cells (Fig. 2b), with clear puncta located on mitotic chromosomes. BiFC-TALE labeled alpha-satellite loci were tracked in HeLa cells (Supplementary Movie S2).

The specificity and efficiency of BiFC-TALE alpha-satellite labeling was verified by immunofluorescence (Fig. 2c). The results showed that the number of centromere puncta labeled in each cell by the BiFC-TALE system was consistent with that by immunofluorescence (Supplementary Fig. S3c,d). Furthermore, quantitative analysis of co-labeled cells showed >85% co-localization (n = 68), with 10 examples of them presented (Fig. 2d), indicating comparable efficiency and specificity. We also demonstrated that the BiFC-TALE-L6/R6 pair worked for centromere labeling in MBA-MD-231 cells and RPE cells (Supplementary Fig. S4).

### BiFC-TALE significantly increased the SBR, compared to full-length FP-based labeling methods

**Figure 3:**
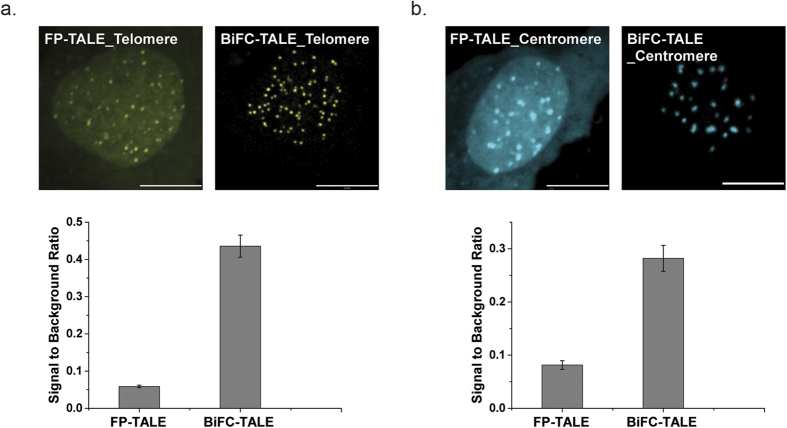
Comparison of BiFC-TALE imaging system and full-length FP-TALE in labeling human telomeres and centromeric alpha-satellites. (**a**) mVenus-based FP-TALE and mVenus-based BiFC-TALE for telomere imaging in living HeLa cells. Cells were transfected either with full-length mVenus-TALE telomere plasmids (upper left) or with mVenus-based BiFC-TALE telomere plasmids (upper right). Scale bars, 10 μm. Lower panel, signal-to-background ratios of both cells, n = 106, error bars are standard error. (**b**) mCerulean-based FP-TALE and mCerulean-based BiFC-TALE for centromeric alpha-satellite imaging in living HeLa cells. Cells were transfected either with full-length mCerulean-TALE alpha-satellite plasmids (upper left) or with mCerulean-based BiFC-TALE alpha-satellite plasmids (upper right). Scale bars, 10 μm. Lower panels, signal-to-background ratios of both cells, n = 56, error bars are standard error.

A key problem of existing approaches to labeling genomic loci is the high background fluorescence, which is largely caused by non-targeted fluorescent protein molecules. The BiFC-TALE system introduces the BiFC technique to genomic sequence labeling, thereby greatly reducing the background fluorescence and increasing the SBR. To determine how much BiFC-TALE actually improves the SBR in practice, we labeled telomeres and centromeres using both BiFC-TALE and the corresponding full-length FP-TALE. Specifically, we used mVenus-based FP-TALE and mVenus-based BiFC-TALE to label telomeres, while using mCerulean-based FP-TALE and mCerulean-based BiFC-TALE to label centromeric alpha-satellites. Significant improvement of SBR was achieved in both cases: the SBR of BiFC-TALE for telomere labeling was ~8-fold higher than that for FP-TALE (Fig. 3a) and 3-fold higher for satellite labeling (Fig. 3b).

To make a direct comparison between BiFC-TALE and FP-TALE, we co-expressed the two labeling systems to label telomeres in the same living cells by co-transfecting HeLa cells with both mVenus-based BiFC-TALE and mCerulean-based FP-TALE with comparable plasmid levels (Supplementary Fig. S6a). Two typical co-localization scenarios for the labeled telomere puncta are presented in Supplementary Fig. S6b, showing that some puncta can be labeled with BiFC-TALE but not with FP-TALE system (Supplementary Fig. S6b Puncta#1) and the SBR of puncta labeled with BiFC-TALE system was higher than FP-TALE system (Supplementary Fig. S6b Puncta#1 and Puncta#2). Quantitative analysis showed that not only was the SBR of the BiFC-TALE system improved >3 times (Supplementary Fig. S6c), but also and more importantly, the number of telomere puncta labeled by the BiFC-TALE system was almost double that of the FP-TALE system (Supplementary Fig. S6d). Moreover, further analysis of BiFC-TALE system and TRF2-mcherry showed that the SBR of BiFC-TALE for telomere labeling was ~5-fold higher than that for TRF2 (Supplementary Fig. S7).

In summary, BiFC-TALE provides more reliable performance with significantly improved SBR for genomic loci labeling in living human cells than the FP-based imaging system,

### Dual-color labeling of telomeres and centromeres in living cells using BiFC-TALE

**Figure 4:**
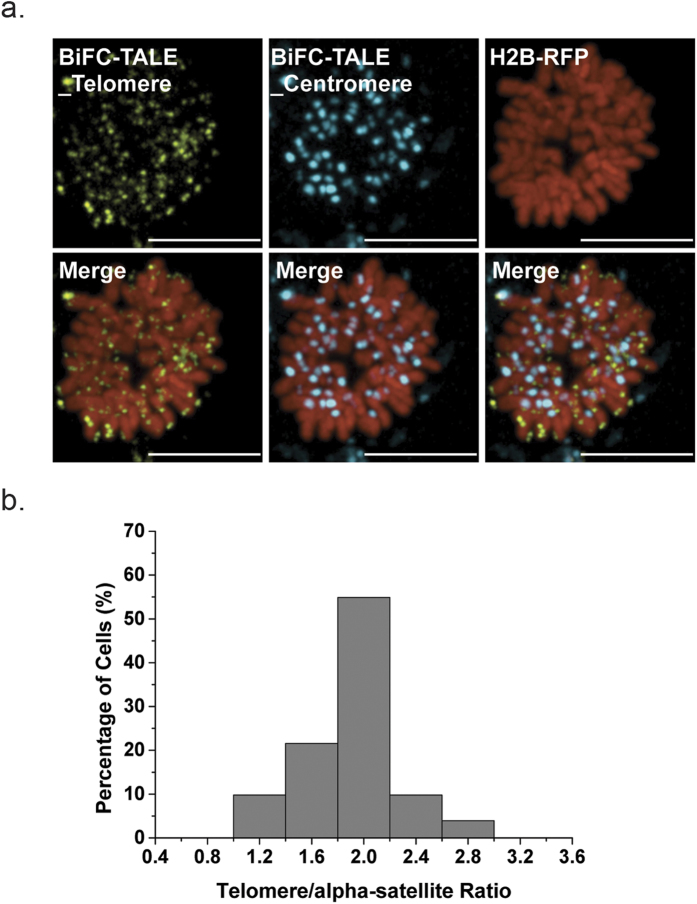
Two-color BiFC-TALE system for visualizing telomere and centromeric alpha-satellite sequences in living human cells. (**a**) BiFC-TALE imaging of telomeres and satellite sequences in mitotic phase HeLa cells: telomeres labeled with BiFC-TALE-L1/R1 (yellow), centromeres labeled with BiFC-TALE-L6/R6 (cyan), and nuclei stained with H2B-RFP (red). Scale bars, 10 μm. (**b**) Quantification of telomere:satellite ratio from two-color BiFC-TALE images of HeLa cells (n = 51).

As many studies require the labeling of multiple genomic loci, we further used BiFC-TALE to create dual-color images by labeling human cell telomeres and centromeres. HeLa cells were co-transfected with BiFC-TALE-L1/R1 plasmids for telomeres and BiFC-TALE-L6/R6 plasmids for centromeric alpha-satellites, then synchronized to the mitotic phase 6 h and imaged 24 h after transfection (Fig. 4a). To estimate the dual-color labeling efficiency of our system, we analyzed the distribution of the telomere:centromere ratio in each cell (Fig. 4b), and found a peak at 2:1 as expected.

## Discussion

The architecture and spatiotemporal organization of chromosomes is essential in biological processes and it would be of great help to have tools for the visualization of specific genomic loci in living cells. While some approaches based on full-length FP have been developed in attempts to realize this goal, their performance is unsatisfactory.

Here, we developed an efficient genomic loci imaging system by combining BiFC and TALE technologies. Since BiFC dramatically increased the SBR by reducing the background fluorescence caused by diffuse full-length FPs, this method enabled the multi-color labeling and tracking of repetitive sequences in living human cells.

Since BiFC-TALE is based on the complementation and reconstitution of fluorescence as two non-fluorescent fragments of a fluorescent protein are brought together, several principles require serious consideration when designing BiFC-TALE probes. The main factors that need to be considered include the spacer length between a BiFC-TALE pair, binding affinity of TALE modules, linker length, and relative orientation of the BiFC-TALE pair. To obtain TALE modules with high DNA binding affinity, TALE design software was recommended. For loci visualization with BiFC-TALE system, the recommended spacer length would be 15–17 bp (BiFC-L1/R1: SL = 17 bp, BiFC-L3/R1: SL = 16 bp, BiFC-L6/R6: SL = 15 bp). Furthermore, given the helical nature of double-stranded DNA, the BiFC-TALE pair may be located on the opposite side of the DNA, depending on the DNA length between each pair. Specifically, as we placed BiFC-TALE pairs on different strands of the DNA, in order to have the pair on the same side of the DNA in a helical shape, the spacer length between the pair would be (5 + 10 m, m = 0, 1, 2…) bp. Our alpha-satellite labeling results indicated that having the BiFC-TALE pair located on the same side of the DNA (BiFC-TALE-L6/R6, Fig. 2b) favors complementation of the BiFC fragments.

Although we have demonstrated that BiFC-TALE is an excellent approach to labeling genomic sequences, it must be noted that the assembly of TALE modules is still quite time-consuming. CRISPR-based BiFC could be another strategy for labeling genomic loci. However, the requirement for protospacer adjacent motif (PAM) sites makes it difficult to implement a paired CRISPR strategy.

Labeling non-repetitive genomic loci is one of the most challenging tasks in live cell chromosomal imaging. The major difficulty is the requirement of co-transfection of large amounts of different DNA-targeting plasmids to provide sufficient signals. As our BiFC-TALE system markedly improved the SBR, the number of plasmids required for non-repetitive genomic loci labeling can be reduced, increasing the labeling efficiency. However, it is still challenging to label non-repetitive sequences with BiFC-TALE system. BiFC-TALE system use plasmid pairs to realize sequences labeling and many different pairs of BiFC-TALE plasmids are required for non-repetitive loci labeling. Since TALE plasmids are big sized, the transient transfection efficiency is low, especially to co-transfect enough BiFC-TALE pairs into the same cell simultaneously in high dosage. What’s more, transient transfection has a shortcoming of rapid plasmid degradation over time, which leads to a short time window for enough plasmids existing in the same cell simultaneously in high dosage. Since lenti-virus based delivery would have recombination problem which can’t be used for BiFC-TLAE system, so far it is not achievable to label single-genomic-loci with this system and we are still trying to find ways to figure the transfection efficiency problem out.

On the whole, our approach provides a powerful imaging system for genomic sequence labeling in living cells, and is an important complement to established genomic loci imaging systems. The remarkable increase of SBR with BiFC technology makes it a promising candidate for single-gene-locus visualization in living cells.

## Materials and Methods

### Construction of telomere BiFC-TALE and centromeric alpha-satellite BiFC-TALE plasmids

The design and assembly of the BiFC-TALE constructs were based on our ULtiMATE protocol^33^. We fused pairs of designed TALE modules with split monomeric Venus (VN173/VC155) for labeling telomeric sequences and monomeric Cerulean (CrN173/CC155)^22^ for centromeric sequences (Supplementary Fig. S1a). The binding sequences we used for telomeres and centromeric alpha-satellites are listed in Supplementary Table S1 and all the amino acid sequences of fluorescent proteins used are listed in Supplementary Sequences. We selected 2 × GGGGS as the linker between TALEs and fluorescent proteins or BiFC non-fluorescent fragments.

### Cell culture, transfection, synchronization, immunofluorescence, and FISH labeling

HeLa and MDA-MB-231 cells were maintained in Dulbecco’s modified Eagle’s medium (DMEM), 10% fetal bovine serum (FBS), 1× penicillin/streptomycin (all reagents from Life Technology). Human retinal pigment epithelium (RPE) cells were maintained in DMEM/F12 with GlutaMAX1, 10% FBS, 1× penicillin/streptomycin (all reagents from Life Technology). All cells were maintained at 37 °C and 5% CO_2_ in a humidified incubator. All plasmids were transfected with FuGENE (Promega) following the manufacture’s recommended protocol.

To synchronize BiFC-TALE labeled HeLa cells to the mitotic phase, starting 6 h after transfection, the cells were treated with thymidine (final concentration, 2 mM; Sigma) for 23 h and then released for 4 h before treatment with Nocodazole (final concentration, 50 nM; Sigma) for 10 h. Then, the synchronized cells were imaged and tracked.

For immunofluorescence, cells were briefly washed twice with phosphate buffered saline (PBS) and incubated twice with extraction buffer (0.5% TritonX-100 in PBS) for 30 s to extract diffuse cytoplasmic proteins. Cells were then washed once with PBS, fixed with 4% paraformaldehyde (PFA) for 15 min at room temperature and then incubated in blocking buffer (0.3% Triton X-100 and 5% BSA in PBS) for 30 min. The cells were then stained with human anti-CREST antibodies (Antibodies Incorporated, #15–235-F) in PBS with 5% BSA overnight, washed with PBS and then stained with Cy5-labeled secondary antibodies in blocking buffer for 1 h. The labeled cells were washed with PBS, and post-fixed with 4% PFA for 10 min at room temperature.

For FISH, BiFC-TALE labeled cells were first fixed with 4% PFA, then washed three times with PBS for 5 min. Then the cells were permeabilized with 0.5% NP-40 in PBS for 30 min and washed with 2 × SSC buffer. The cells were incubated with 100 μg/ml RNase A at 37 °C for 1 h and then washed with 2 × SSC buffer. Then, the cells were dehydrated with 70% ethanol for 5 min, 85% ethanol for 5 min, and 100% ethanol for 5 min. The cells were denatured with 70% formamide in 2 ×SSC buffer at 80 °C for 10 min.

### Image acquisition

All images were captured by Volocity software (PerkinElmer) on an inverted TiE microscope (Nikon) attached to an UltraVIEW VOX system spinning-disc (PerkinElmer) with an EMCCD camera (Hamamatasu, C9100-13). For imaging, cells were grown on 35-mm glass-bottomed dishes. The microscope stage incubation chamber was maintained at 37 °C and 5% CO_2_. mVenus, mCerulean, RFP and DAPI were excited with 514-nm (25 mW), 440-nm (40 mW), 561 nm (50 mW) and 405 nm (50 mW) lasers, respectively. All channels were collected with a N.A. 1.25 TIRF 100×oil objective (Nikon CFI APO). An 8-μm z-stack of all channels with a step-size of 0.4 μm was acquired for all images. In the long-term time-lapse tracking of cell division, an 8-μm z-stack of both channels with a step-size of 0.4 μm was acquired every 15 min for ~12 h. To reduce the photo-damage effect on the cells, all 405, 561, and 514 nm lasers were set to 10% transmission.

### Image analysis

#### SBR calculation

Background and signal intensities were quantified using ImageJ, and then the signal-to-background ratio was calculated as follows: SBR = (I-B)/B, where I is the intensity of the area of interest (puncta labeled) and B is the intensity of the background signal. To be specific, we first measured the intensity of puncta and then chose five points around it as the background. The average intensity of these five points was calculated as the intensity of the background signal.

#### FISH images analysis

Since HeLa cells will become a little shrinking after FISH procedure, we used the Registering an Image:: Imaging Registration (Image Processing Toolbox) from MatLab to align the images, following the recommended procedure. This imaging tool can helps overcome issues such as image rotation, scale, and skew, which are common when overlaying images. Co-localization of telomere BiFC-TALE and telomere FISH images was analyzed after that.

#### Co-localization analysis

Images of a z stack were projected into a 2D image by maximum intensity projection and then converted into 8 bit images. For each channel images, we first chose the menu item “Analyze-Analyze Particles” to set appropriate threshold and count puncta numbers. Co-localization analysis was carried out with the Image J plugin “colocalization” with threshold obtained in the first step. More than 50% overlay of puncta from the two channels is identified as co-localization (Supplementary Fig. S8).

## Additional Information

**How to cite this article**: Hu, H. *et al*. Live visualization of genomic loci with BiFC-TALE. *Sci. Rep.* **7**, 40192; doi: 10.1038/srep40192 (2017).

**Publisher's note:** Springer Nature remains neutral with regard to jurisdictional claims in published maps and institutional affiliations.

## Supplementary Material

Supplementary Information

Supplementary Movie S1

Supplementary Movie S2
