# Supplementary material for: Live visualization of genomic loci with BiFC-TALE
Source: Sci Rep. 2017 Jan 11;7:40192. doi: 10.1038/srep40192 (PMC5225478; doi:10.1038/srep40192)
Supplement: Supplementary Information [file srep40192-s1.pdf]

Live visualization of genomic loci with BiFC-TALE

Huan Hu[1, 2, +], Hongmin Zhang[1, 3, 4, 5, +], Sheng Wang[1, 4, +], Miao Ding[1, 4], Hui An[1, 2], Yingping Hou[1, 4], Xiaojing Yang[2], Wensheng Wei[1, 3, 4, 6, 7, *], Yujie Sun[1, 4, *], Chao Tang[2, 3, *]

[1]School of Life Sciences, Peking University, Beijing 100871, China. [2]Center for Quantitative Biology, Peking University, Beijing 100871, China. [3]Peking-Tsinghua Center for Life Sciences, Peking University, Beijing 100871, China. [4]Biodynamic Optical Imaging Center (BIOPIC), Peking University, Beijing 100871, China. [5]Academy for Advanced Interdisciplinary Studies, Peking University, Beijing 100871, China. [6]Beijing Advanced Innovation Center for Genomics (ICG), Peking University, Beijing 100871, China. [7]State Key Laboratory of Protein and Plant Gene Research, Peking University, Beijing 100871, People's Republic of China.

[*]Email: tangc@pku.edu.cn, sun_yujie@pku.edu.cn, wswei@pku.edu.cn

[+]these authors contributed equally to this work

SUPPLEMENTARY FIGURES

a.

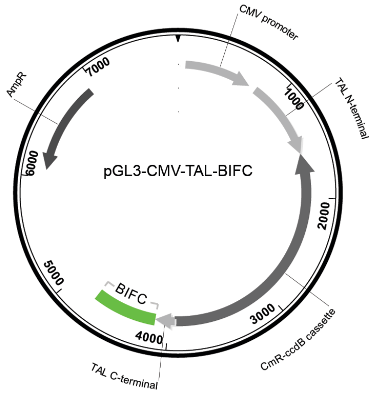

b.

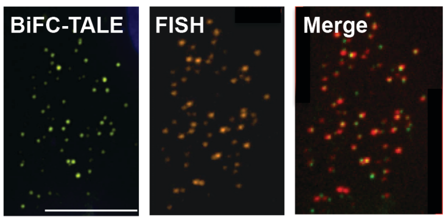

c.

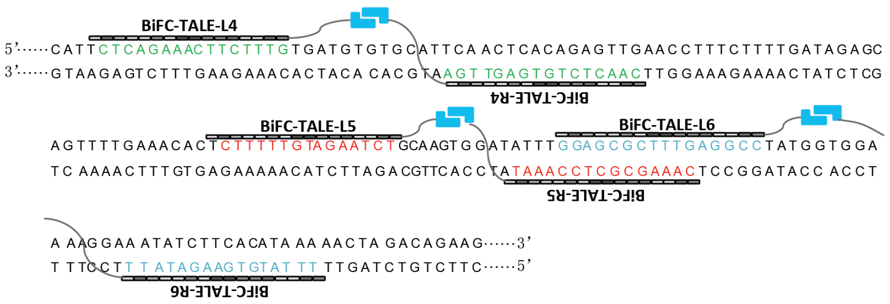

Supplementary Figure S1. (a) Backbone plasmid for BiFC-TALE system. (b) Example cell of telomeres co-labeled using BiFC-TALE (yellow) and FISH (red). Scale bar, 10 μm. (c) Binding of BiFC-TALE-L4/R4, L5/R5 and L6/R6 on alpha-satellite 171-bp repeat.

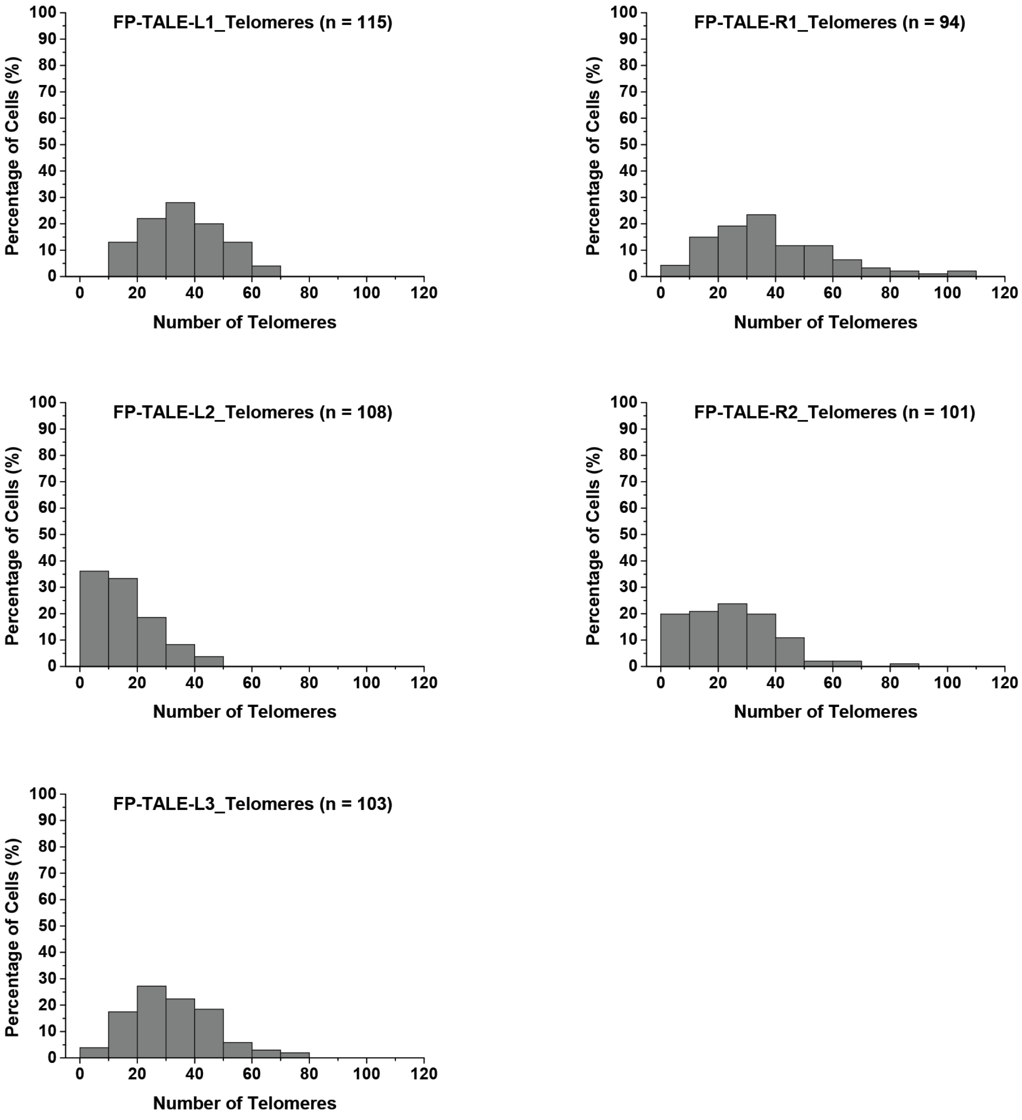

Supplementary Figure S2. Histograms of the number of detected telomeres by FP-TALE-L1, FP-TALE-L2, FP-TALE-L3, FP-TALE-R1 and FP-TALE-R2 in living human cells.

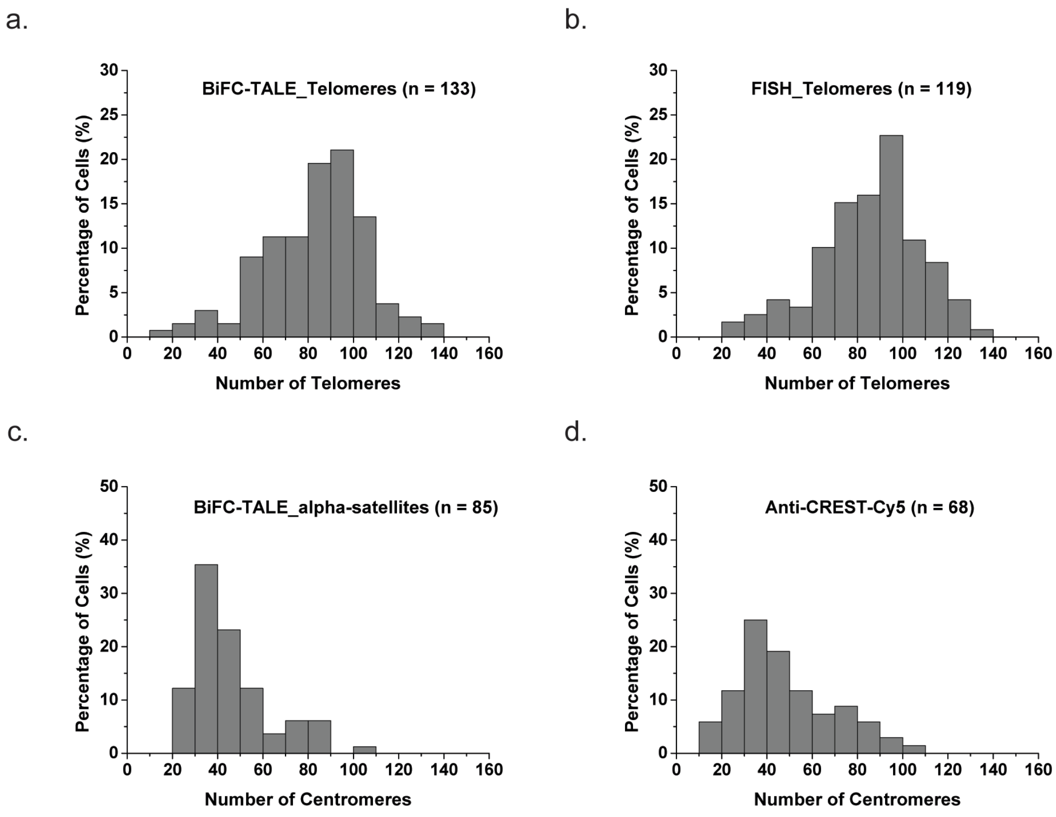

Supplementary Figure S3. Histograms of telomeres and centromeric alpha-satellites labeled in living human cells. (a) Histogram of the number of detected telomeres by BiFC-TALE, n=133. (b) Histogram of the number of detected telomeres by FISH, n=119. (c) Histogram of the number of detected centromeric alpha-satellites by BiFC-TALE, n=85. (d) Histogram of the number of detected centromeric alpha-satellites by IF, n=68.

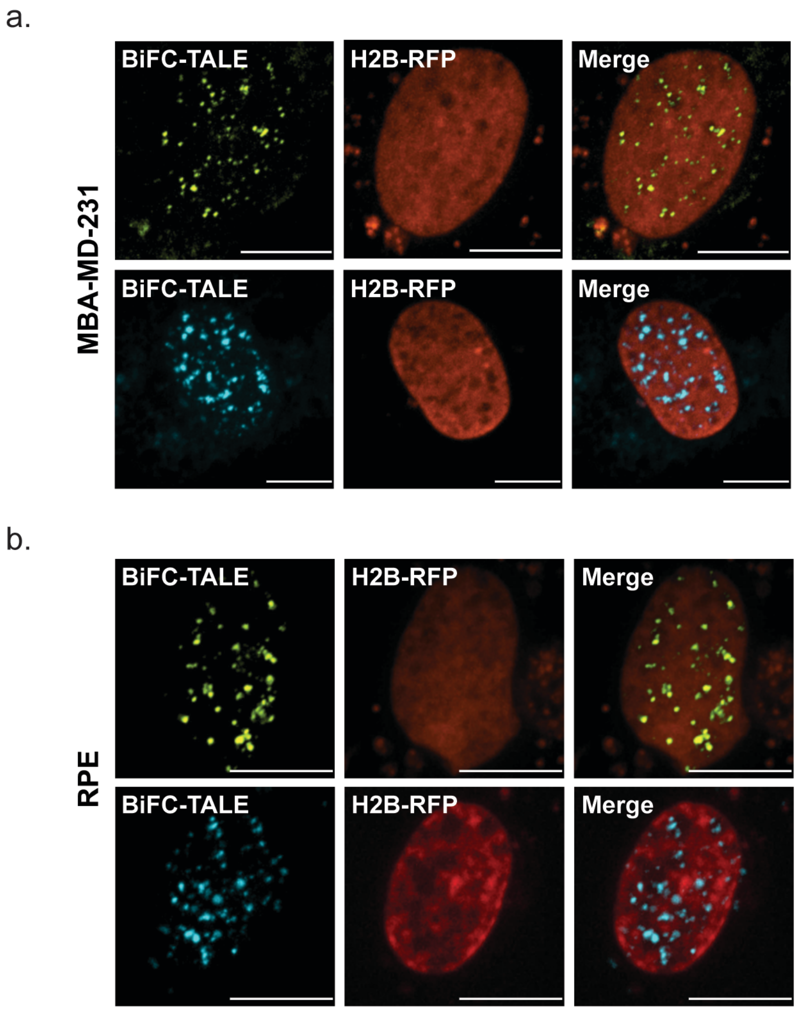

Supplementary Figure S4. BiFC-TALE imaging of telomeres and centromeric alpha-satellites in living MBA-MD-231 cells and RPE cells. (a) BiFC-TALE imaging of telomeres (yellow) and alpha-satellites (cyan) in MBA-MD-231 cells; nuclei stained with H2B-RFP (red). Scale bars, 10 μm. (b) BiFC-TALE imaging of telomeres (yellow) and alpha-satellites (cyan) in RPE cells; nuclei stained with H2B-RFP (red). Scale bars, 10 μm.

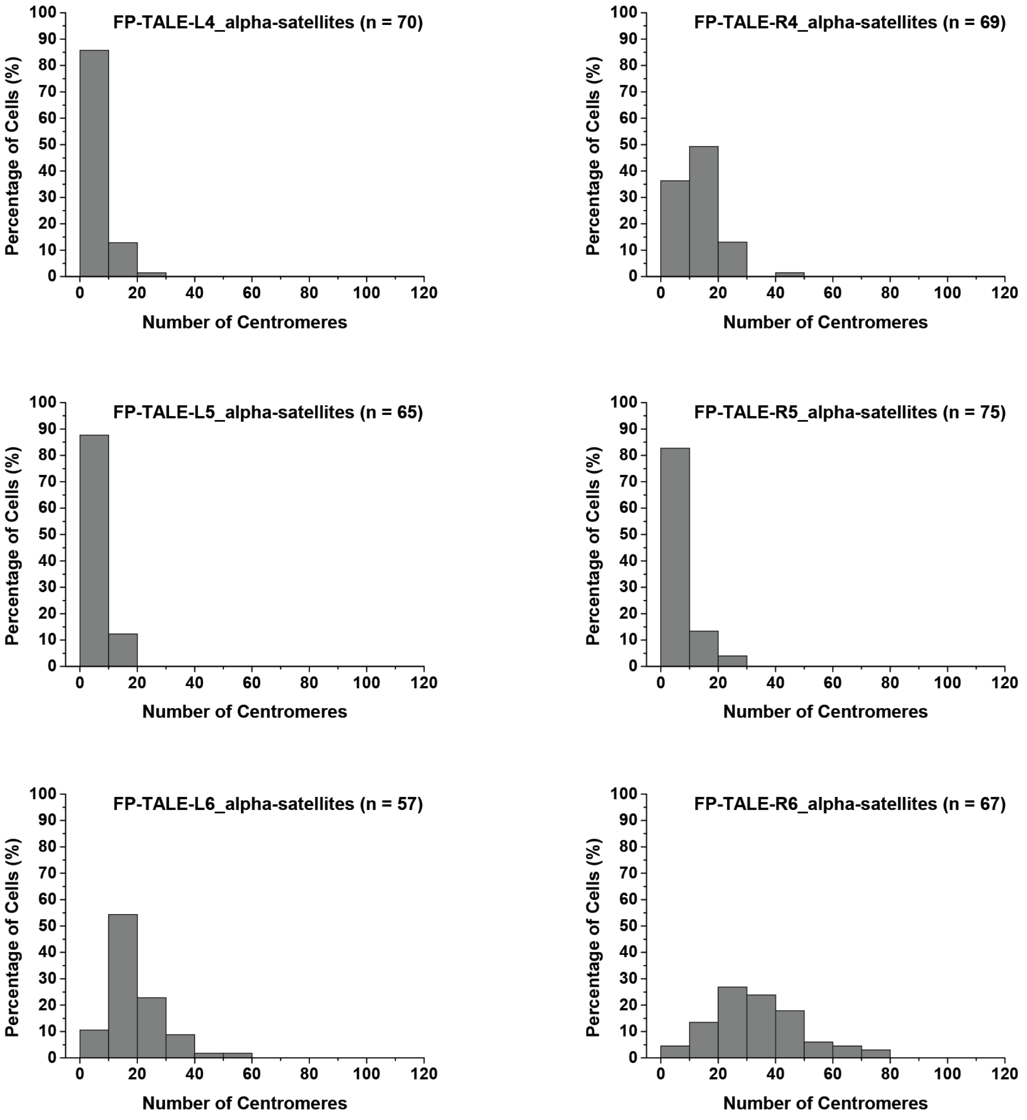

Supplementary Figure S5. Histograms of the number of detected alpha-satellites by FP-TALE-L4, FP-TALE-L5, FP-TALE-L6, FP-TALE-R4, FP-TALE-R5 and FP-TALE-R6 in living human cells.

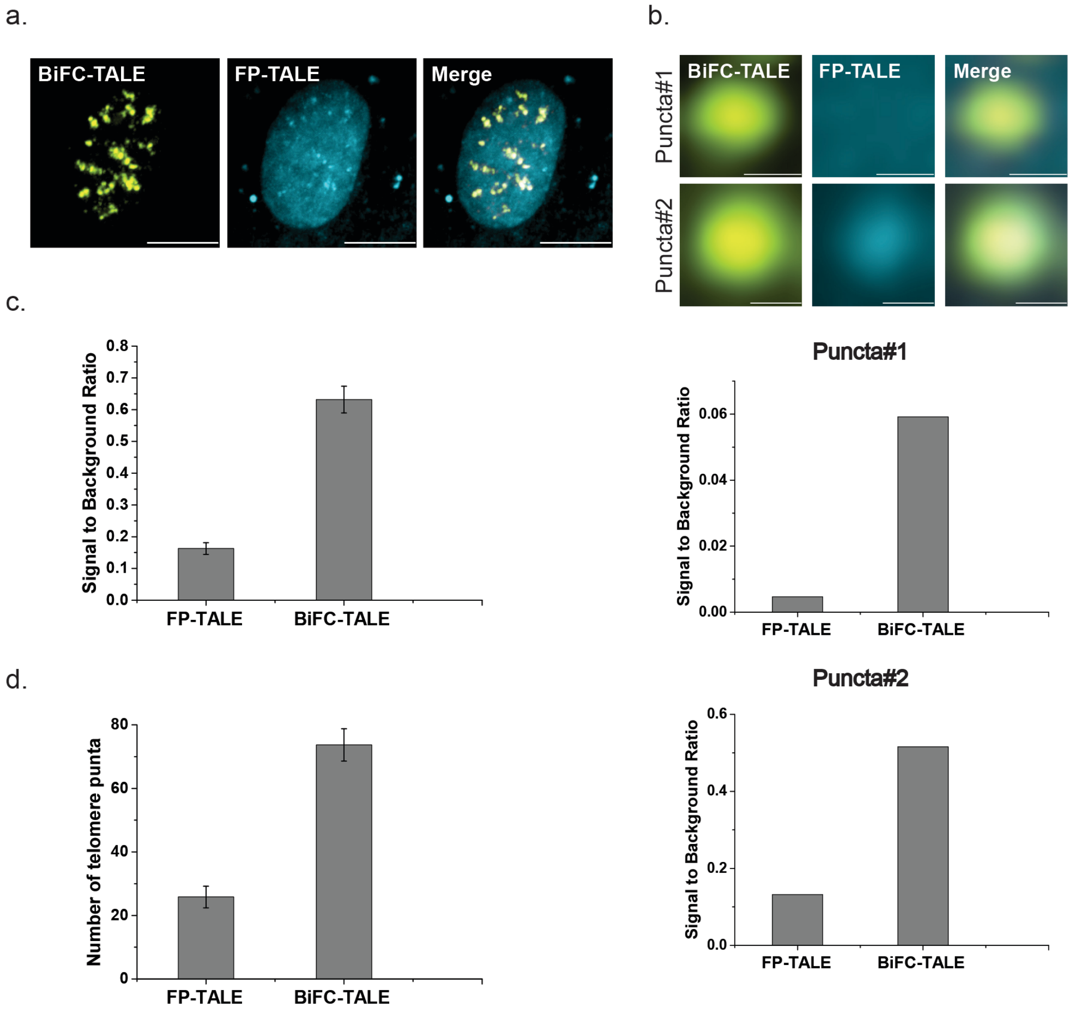

Supplementary Figure S6. Comparison of BiFC-TALE imaging system and full-length FP-TALE by labeling human telomeres in HeLa cells. (a) BiFC-TALE-L1/R1 (yellow) plasmids were co-expressed with mCerulean-based FP-TALE (cyan) plasmid for telomere sequences labeling in living HeLa cells. Scale bars, 10 μm. (b) Two typical co-localization scenarios of BiFC-TALE and FP-TALE labeled telomere puncta. Scale bars, 0.5 μm. Quantitatively analyzed SBR of them were shown below. (c) The signal-to-noise ratios of BiFC-TALE and FP-TALE labeled puncta in cells were calculated, n=73. Error bars are standard error. (d) Quantification of the number of telomere puncta in BiFC-TALE and FP-TALE labeled cells, n=73. Error bars are standard error.

a.

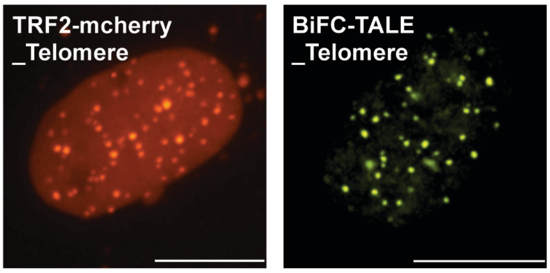

b.

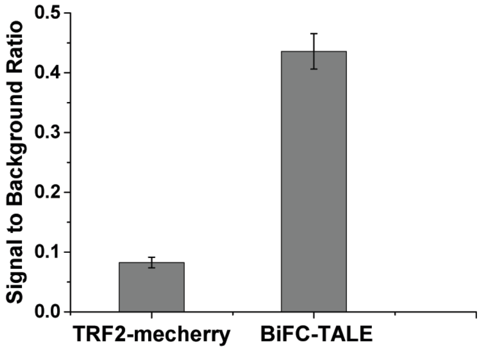

Supplementary Figure S7. Comparison of TRF2-mcherry and BiFC-TALE imaging system by labeling human telomeres. (a) Comparison between TRF2-mcherry and mVenus-based BiFC-TALE for telomeres imaging in living HeLa cells. HeLa cells were transfected either TRF2-mcherry Telomere plasmid (up, left) or mVenus-based BiFC-TALE Telomere plasmids (up, right). Scale bars, 10 μm. (b) The signal-to-noise ratios of both cells were calculated (bottom), (n=106, n = 95). Error bars are standard error.

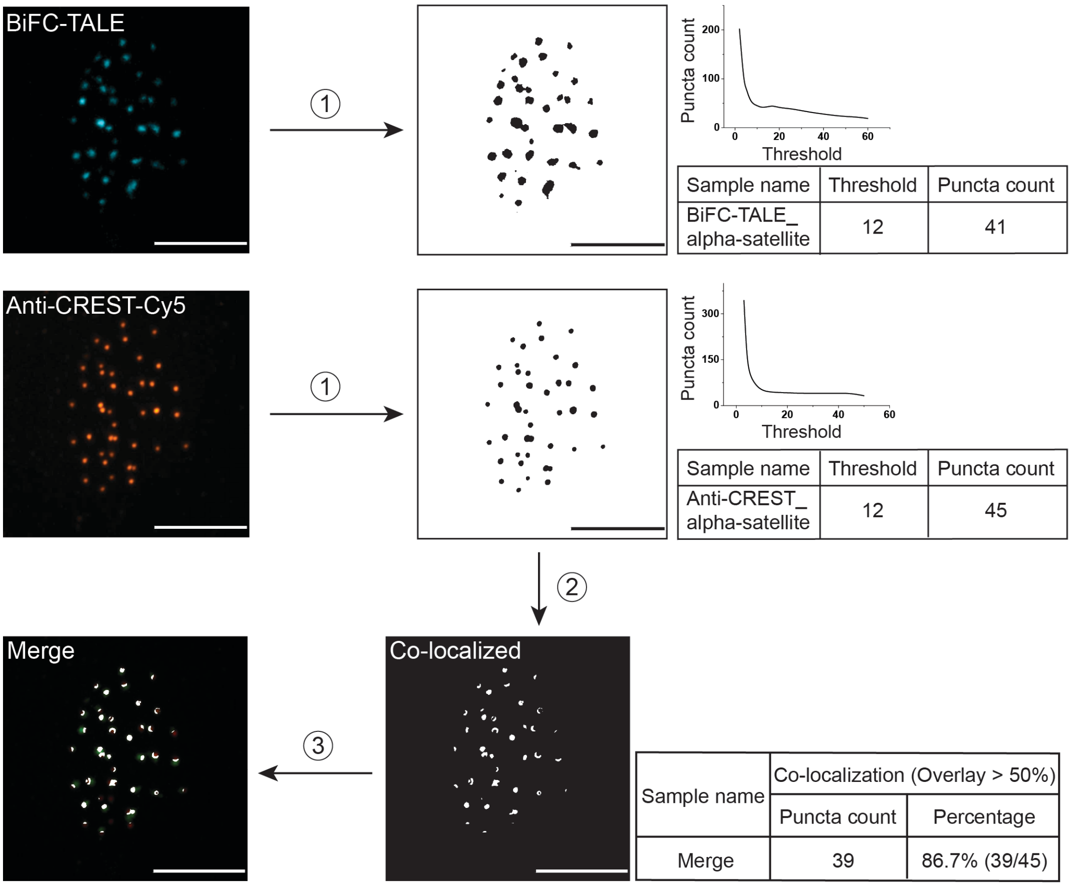

Sample name	Threshold	Puncta count
BiFC-TALE_ alpha-satellite	12	41

Sample name	Threshold	Puncta count
Anti-CREST_ alpha-satellite	12	45

Sample name	Co-localization (Overlay > 50%)	
	Puncta count	Percentage
Merge	39	86.7% (39/45)

Supplementary Figure S8. Example of imageJ co-localization analysis with BiFC-TALE and IF co-labeled HeLa cell images. Step1: Previously projected images from each channel were converted into 8 bit images before we chose the menu item "Analyze-Analyze Particles" in imageJ to set appropriate threshold and count puncta numbers. Step2: Co-localization analysis was carried out with the Image J plugin "colocalization" with threshold obtained in the first step. More than 50% overlay of puncta from the two channels is identified as co-localization with co-localized dots counted and co-localization percentage calculated. Step3: Images from the two channels were merged with the co-localized image obtained in step2.

SUPPLEMENTARY TABLES

Supplementary Table S1. BiFC-TALE and FP-TALE plasmids constructed for targeted DNA sequences in human chromosomes.

BIFC-TALE plasmid	Targeted sequence	BiFC fragments	Repetitive DNA sequences
BiFC-TALE-L1	TAGGGTTAGGGTTAGG	VN173 and VC155	Telomere
BiFC-TALE-L2	TAGGGTTAGGGTTA		
BiFC-TALE-L3	TAGGGTTAGGGTTAGGG		
BiFC-TALE-R1	AACCCTAACCCTAACC		
BiFC-TALE-R2	AACCCTAACCCTAACCC		
BiFC-TALE-L4	CTCAGAAACTTCTTTG	CrN173 and CC155	Alpha-satellite
BiFC-TALE-L5	CTTTTTGTAGAATCT		
BiFC-TALE-L6	GGAGCGCTTTGAGGCC		
BiFC-TALE-R4	CAACTCTGTGAGTTGA		
BiFC-TALE-R5	CAAAGCGCTCCAAAT		
BiFC-TALE-R6	TTTATGTGAAGATATT		

FP-TALE plasmid	Targeted sequence	Fluorescent protein (FP)	Repetitive DNA sequences
FP-TALE-L1	TAGGGTTAGGGTTAGG	mVenus	Telomere
FP-TALE-L2	TAGGGTTAGGGTTA		
FP-TALE-L3	TAGGGTTAGGGTTAGGG		
FP-TALE-R1	AACCCTAACCCTAACC		
FP-TALE-R2	AACCCTAACCCTAACCC		
FP-TALE-L1	TAGGGTTAGGGTTAGG	mCerulean	
FP-TALE-L4	CTCAGAAACTTCTTTG	mCerulean	Alpha-satellite
FP-TALE-L5	CTTTTTGTAGAATCT		
FP-TALE-L6	GGAGCGCTTTGAGGCC		
FP-TALE-R4	CAACTCTGTGAGTTGA		
FP-TALE-R5	CAAAGCGCTCCAAAT		
FP-TALE-R6	TTTATGTGAAGATATT		

Supplementary Table S2. Combinations of BiFC-TALE pairs with different space length and different binding sites for telomere and alpha-satellite sequences.

TALE-BIFC pair combination	Spacer Length (SL)	Repetitive DNA sequences
L1+R1	(5+6n) bp	Telomere
L1+R2	(4+6n) bp	
L2+R1	(7+6n) bp	
L2+R2	(6+6n) bp	
L3+R1	(4+6n) bp	
L3+R2	(3+6n) bp	
L4+R4	12 bp	Alpha-satellite
L5+R5	10 bp	
L6+R6	15 bp	

SUPPLEMENTARY SEQUENCES

a. Full-length mVenus:

MVSKGEELFTGVVPILVELDGDVNGHKFSVSGEGEGDATYGKLTLKFICTTGKLPVPWPTLV
TTFGYGLQCFARYPDHMKQHDFFKSAMPEGYVQERTIFFKDDGNYKTRAEVKFEGDTLVNR
IELKGIDFKEDGNILGHKLEYNYNSHNVYIMADKQKNGIKVNFKIRHNIEDGSVQLADHYQQN
TPIGDGPVLLPDNHYLSYQSALSKDPNEKRDHMVLLEFVTAAGITLGMDELYK

b. Split mVenus fragment (VN173):

MVSKGEELFTGVVPILVELDGDVNGHKFSVSGEGEGDATYGKLTLKLICTTGKLPVPWPTLV
TTLGYGLQCFARYPDHMKQHDFFKSAMPEGYVQERTIFFKDDGNYKTRAEVKFEGDTLVNR
IELKGIDFKEDGNILGHKLEYNYNSHNVYITADKQKNGIKANFKIRHNIE

c. Split mVenus fragment (VC155):

DGGVQLADHYQQNTPIGDGPVLLPDNHYLSYQSALSKDPNEKRDHMVLLEFVTAAGITLGM
DELYK

d. Full-length mCerulean:

MVSKGEELFTGVVPILVELDGDVNGHKFSVSGEGEGDATYGKLTLKFICTTGKLPVPWPTLV
TTLTWGVQCFARYPDHMKQHDFFKSAMPEGYVQERTIFFKDDGNYKTRAEVKFEGDTLVN
RIELKGIDFKEDGNILGHKLEYNAISDNVYITADKQKNGIKANFKIRHNIEDGSVQLADHYQQN
TPIGDGPVLLPDNHYLSTQSKLSKDPNEKRDHMVLLEFVTAAGITLGMDELYK

e. Split mCerulean (CrN173):

MVSKGEELFTGVVPILVELDGDVNGHKFSVSGEGEGDATYGKLTLKFICTTGKLPVPWPTLV
TTLTWGVQCFARYPDHMKQHDFFKSAMPEGYVQERTIFFKDDGNYKTRAEVKFEGDTLVN
RIELKGIDFKEDGNILGHKLEYNAISDNVYITADKQKNGIKANFKIRHNIE

f. Split mCerulean (CC155):

DKQKNGIKANFKIRHNIEDGSVQLADHYQQNTPIGDGPVLLPDNHYLSTQSKLSKDPNEKRD
HMVLLEFVTAAGITLGMDELYK

SUPPLEMENTARY MOVIES

Supplementary Movie S1. Live imaging of telomeres in mitotic HeLa cell using BiFC-TALE. The cell became out of focus after division. Scale bar, 10 µm.

Supplementary Movie S2. Live imaging of alpha-satellites in HeLa cell using BiFC-TALE. Scale bar, 10 µm.
